# Characterisation and application of a bovine U6 promoter for expression of short hairpin RNAs

**DOI:** 10.1186/1472-6750-5-13

**Published:** 2005-05-11

**Authors:** Luke S Lambeth, Robert J Moore, Morley Muralitharan, Brian P Dalrymple, Sean McWilliam, Timothy J Doran

**Affiliations:** 1 CSIRO Livestock Industries, Australian Animal Health Laboratory, Geelong, VIC 3220, Australia; 2 School of Biological and Chemical Sciences, Deakin University, Geelong, VIC 3217, Australia; 3 CSIRO Livestock Industries, Queensland Bioscience Precinct, St Lucia, QLD 4067, Australia

## Abstract

**Background:**

The use of small interfering RNA (siRNA) molecules in animals to achieve double-stranded RNA-mediated interference (RNAi) has recently emerged as a powerful method of sequence-specific gene knockdown. As DNA-based expression of short hairpin RNA (shRNA) for RNAi may offer some advantages over chemical and *in vitro *synthesised siRNA, a number of vectors for expression of shRNA have been developed. These often feature polymerase III (pol. III) promoters of either mouse or human origin.

**Results:**

To develop a shRNA expression vector specifically for bovine RNAi applications, we identified and characterised a novel bovine U6 small nuclear RNA (snRNA) promoter from bovine sequence data. This promoter is the putative bovine homologue of the human U6-8 snRNA promoter, and features a number of functional sequence elements that are characteristic of these types of pol. III promoters. A PCR based cloning strategy was used to incorporate this promoter sequence into plasmid vectors along with shRNA sequences for RNAi. The promoter was then used to express shRNAs, which resulted in the efficient knockdown of an exogenous reporter gene and an endogenous bovine gene.

**Conclusion:**

We have mined data from the bovine genome sequencing project to identify a functional bovine U6 promoter and used the promoter sequence to construct a shRNA expression vector. The use of this native bovine promoter in shRNA expression is an important component of our future development of RNAi therapeutic and transgenic applications in bovine species.

## Background

RNA interference (RNAi), a method of sequence specific gene knockdown, has been used to analyse gene function in plants, invertebrates, and more recently mammalian cells [1-3]. The conserved RNAi pathway involves the processing of double stranded RNA (dsRNA) duplexes into 21–23 nucleotide (nt) molecules known as small interfering RNAs (siRNA) to initiate gene knockdown [4-6]. Since the discovery of RNAi in animals [7] the use of long dsRNA in lower eukaryotes, especially in the model organism *Caenorhabditis elegans*, has been used to determine gene function [8,9]. However, in mammalian systems the cellular uptake of long dsRNA induces an antiviral defence mechanism initiated by interferon (IFN), leading to non-specific translational shutdown and apoptosis [10-12].

This non-specific cellular activity can be circumvented by the direct transfection of either chemically synthesised or *in vitro *transcribed siRNAs of approximately 21 nt in length into mammalian cells [1,13]. These short molecules do not activate the IFN response, but can induce reliable and efficient transient knockdown of target genes [14,15]. As a consequence, the development of DNA-based vectors for expression of short hairpin RNA (shRNA) molecules that are processed within the cell to produce active siRNA molecules has progressed rapidly [16-18]. Such DNA expression constructs have achieved highly efficient gene knockdown without induction of the IFN response.

DNA-based vectors offer some additional advantages over chemical and *in vitro *synthesised siRNA. Vector construction is much less expensive compared to the chemical synthesis of siRNA, selection of transfected cells is possible via antibiotic selection and the option of inducible shRNA transcription is also available. shRNA expression vectors have been engineered using both viral (including retroviral [19], adenoviral [20] and lentiviral [21] vectors), and plasmid systems [16-18]. These vectors often utilize promoters from a small class of pol. III promoters [22,23] to drive the expression of shRNA. Promoters of this type are preferred because they naturally direct the synthesis of small, highly abundant non-coding RNA transcripts, with defined termination sequences consisting of 4–5 thymidines (Ts) and have no requirement for downstream promoter elements [22-24].

The human U6 snRNA promoter is the best studied type III pol. III promoter. It has characteristic promoter elements known as the enhancer and core regions [25,26], and is frequently used in RNAi expression vectors. A total of nine full-length U6 loci have been identified from the human genome [27]. These genes are dispersed throughout the genome and five of these are potentially active, including the previously described human U6 promoter, now denoted U6-1. In the current study we describe the characterisation of the bovine U6 snRNA promoter and its expression of shRNA molecules in bovine cells. We used a PCR based cloning strategy to construct a plasmid vector that features the bovine U6 promoter to drive expression of shRNA molecules directed at the exogenously expressed Enhanced Green Fluorescent Protein (EGFP) and the endogenous bovine gene, glyceraldehyde-3-phosphate dehydrogenase (GAPDH).

## Results and discussion

### Characterisation of a bovine U6 promoter

**Figure 1:**
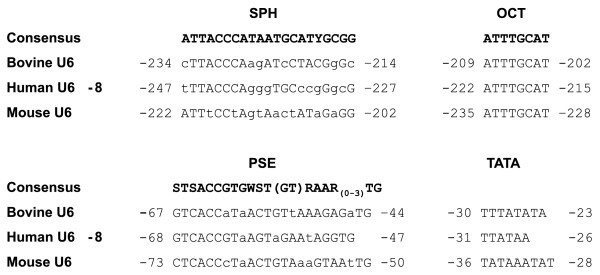
**Promoter element sequences of the bovine U6, human U6-8 and mouse U6 promoters. **The distal promoter regions containing the SPH and OCT sequences and proximal promoter regions containing the PSE and TATA sequence elements are shown for each promoter. Matches to the consensus sequence delineated at the top of the SPH, OCT and PSE sequences are shown in upper case (SPH consensus is from Schaub *et al*., 1999 [28], PSE consensus is from Dahlberg *et al*., 1988 [29] and OCT consensus is from Sturm *et al*. 1988 [30]). Note that the relative positions of the SPH and OCT elements are switched in the mouse promoter.

A bovine BAC clone was identified from GenBank (Accession no. CC528275) that contained a 107 nt region that shared 100% identity with the human U6 snRNA sequence. A region directly upstream of this sequence also shared significant homology with previously identified human U6 promoter sequences. BLAT analysis revealed that this BAC-clone shared the greatest sequence homology with the human U6-8 promoter located on chromosome 14 [27]. The bovine BAC sequence contained upstream promoter elements consistent with the human U6 promoters and other human pol. III promoters [27-30] (Fig. 1). These elements are also present in the mouse U6 promoter used in the p*Silencer *1.0-U6 siRNA Expression Vector (Ambion). The location and spacing of these elements is similar for all human U6 promoters and their requirement for pol. III activity is well documented [28-30]. The presence of these elements in the bovine BAC-clone sequence directly upstream of the full-length U6 snRNA suggested that this region may be an active bovine promoter and could be used effectively to promote shRNA expression.

### shRNA expression vector construction

**Figure 2:**
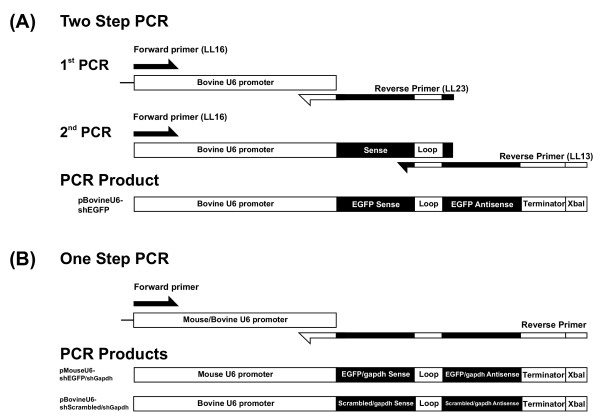
**Schematic representation of the PCR strategies used to produce shRNA expression vectors. **(A): The two-step PCR method used to generate pBovineU6-shEGFP. The 1^st ^PCR amplified the bovine U6 promoter, EGFP shRNA sense, loop, and 3 nt of EGFP shRNA antisense using primers LL16 and LL23. The 2^nd ^PCR amplified the bovine U6 promoter and the remaining EGFP shRNA components including EGFP shRNA antisense, terminator and *Xba*I using primers LL16 and LL13. (B): The one-step PCR method used to generate pMouseU6-shEGFP, pBovineU6-shScrambled, pMouseU6-shGapdh and pBovineU6-shGapdh. PCR reactions used forward primers paired with single reverse primers comprising all shRNA components. All final PCR products consisted of a mouse or bovine U6 promoter, shRNA sense, loop, shRNA antisense, termination sequence and *Xba*I site.

Using the predicted bovine U6 snRNA promoter sequence as template, PCR based cloning strategies were used to generate shRNA expression vectors targeting EGFP and bovine GAPDH. A two-step PCR reaction was used to produce a vector targeting EGFP (pBovineU6-shEGFP) (Fig. 2A). One-step PCR reactions were used to produce an shRNA expression vector targeting GAPDH (pBovineU6-shGapdh), and a non-specific control shRNA (pBovineU6-shScrambled) (Fig. 2B). In addition, using the mouse U6 snRNA promoter sequence from p*Silencer*-1.0 as template in one-step PCR reactions, shRNA expression vectors targeting EGFP (pMouseU6-shEGFP) and bovine GAPDH (pMouseU6-shGapdh) were produced (Fig. 2B).

In all constructs, the first nucleotide of the predicted shRNA was a guanine (G) residue, corresponding to the first nucleotide of the native U6 snRNA. An *Xba*I restriction enzyme site was engineered downstream of the termination signal to allow screening for full-length shRNA products inserted into pGEM-T Easy (Promega) which lacks an *Xba*I site. All final shRNA expression constructs consisted of either the full-length bovine or mouse U6 promoter, a shRNA sense sequence, a loop sequence, a shRNA antisense sequence, a termination sequence and an *Xba*I site.

### Activity of the bovine U6 promoter measured by EGFP knockdown

To analyse the function of the bovine U6 promoter, the level of EGFP expression in cells cotransfected with pBovineU6-shEGFP and pEGFP-N1 (Clonetech) were directly compared with EGFP expression in cells cotransfected with pBovineU6-shScrambled and pEGFP-N1. An indication of the bovine promoter efficiency was provided by direct comparison to the level of EGFP knockdown by the same shRNA molecule expressed from pMouseU6-shEGFP. Prior to validation in bovine cells, both mouse and bovine U6 promoter driven shRNA expression vectors were first validated in Vero cells. As this cell line lacks the interferon α, β and ω genes [31,32], a significant reduction in EGFP expression could be attributed to RNAi and not the result of non-specific inhibition of protein translation characteristic of the IFN response triggered by expressed exogenous dsRNA.

**Figure 3:**
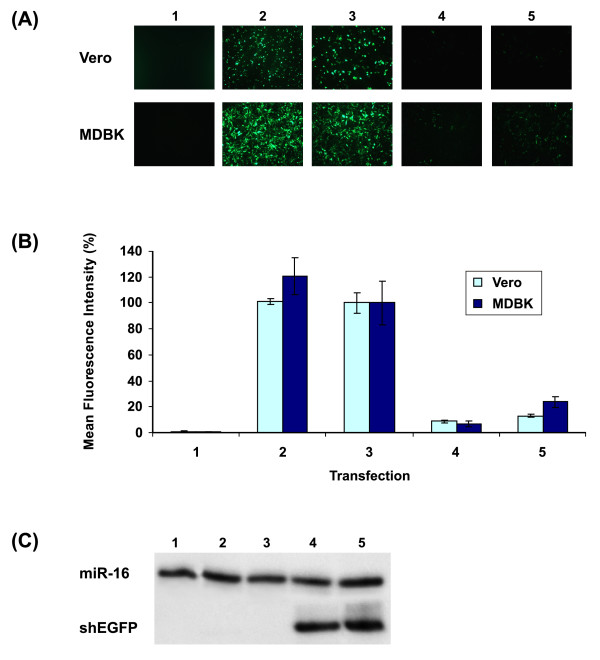
**Inhibition of EGFP expression and detection of expressed shRNA from bovine and mouse U6 promoters. **(1) transfection free negative control (cells only); (2) transfection of pEGFP-N1; (3) cotransfection of pEGFP-N1 and pBovineU6-shScrambled; (4) cotransfection of pEGFP-N1 and mouseU6-shEGFP; (5) cotransfection of pEGFP-N1 and pBovineU6-shEGFP. All transfections were performed in triplicate. (A): Typical fluorescence images of Vero and MDBK cells (Magnifications ×100). (B): Mean Fluorescence Intensity (MFI) of Vero and MDBK cells 72 hours post-transfection determined by flow cytometry. MFI of EGFP expressed as a percentage of the MFI of pEGFP-N1 + pBovineU6-shScrambled. (C): Detection of EGFP shRNAs in Vero cell extracts. Small RNAs were isolated from Vero cells and hybridised to a radiolabelled RNA probe identical in sequence to the EGFP shRNA (shEGFP). shEGFP was detected in lanes 4 and 5 only, and miR-16 was used as a loading control.

For each transfection condition, knockdown of EGFP in Vero and MDBK cell lines was visualised by fluorescence microscopy (Fig. 3A). Flow cytometry was used to determine the Mean Fluorescence Intensity (MFI) (Fig. 3B). Results in both cell lines showed that cells transfected with either pMouseU6-shEGFP or pBovineU6-shEGFP exhibited large reductions in EGFP expression when compared with cells transfected with pBovineU6-shScrambled.

To ensure that the observed reduction in EGFP expression could be directly attributed to RNAi induced by expressed shRNA, the transcription of these molecules was detected in transfected cells. A radiolabelled RNA probe complementary in sequence to the EGFP shRNA sequence was used in an RNAse protection assay to visualise these molecules. The mouse microRNA (miRNA) miR-16 probe used as a loading control for each condition produced a strong signal at the expected size for all samples (Fig. 3C). The EGFP shRNA was detected only in those samples that were transfected with either of the EGFP shRNA vectors (Fig. 3C). There were no obvious differences in the amount of shRNA produced by either the mouse or bovine U6 promoters.

### Activity of the bovine U6 promoter measured by GAPDH knockdown

**Figure 4:**
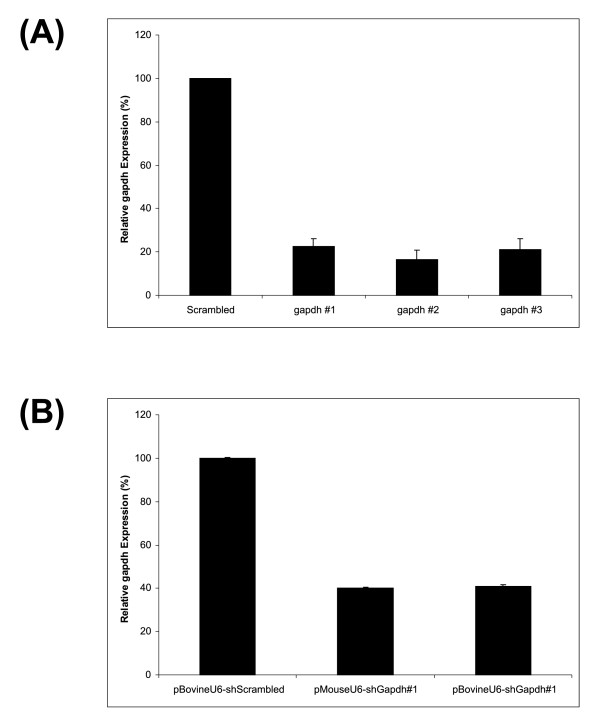
**Inhibition of bovine GAPDH expression in MDBK cells. **The amount of GAPDH in MDBK cells was determined by Real-time PCR 48 hours post-transfection, expressed as a relative percentage compared to the amount of GAPDH in cells transfected with a scramble sequence siRNA. (A): Cells transfected with either siRNAs directed at bovine GAPDH or a scrambled sequence control siRNA. (B): MDBK cells transfected with pBovine-shScrambled, pMouse-shGapdh and pBovine-shGapdh.

To further validate the function of the bovine U6 promoter for RNAi, shRNA expression vectors targeting the endogenous bovine gene GAPDH were produced. An active shRNA sequence was identified by testing three siRNA sequences directed at this gene. Real-time PCR was used to determine the relative amount of GAPDH in MDBK cells transfected with the three siRNAs compared to cells transfected with the scrambled sequence siRNA control. A similar level of knockdown was achieved for each of the bovine GAPDH siRNAs (Fig. 4A). The most appropriate siRNA sequence, GAPDH #1, was selected for use in construction of an shRNA vector as this sequence did not contain any runs of T or A residues that may cause premature termination of expressed shRNAs. This sequence was used for both mouse and bovine U6 shRNA vectors (pMouse-shGapdh and pBovine-shGapdh). Using Real-time PCR, the relative amount of GAPDH in MDBK cells transfected with pMouse-shGapdh and pBovine-shGapdh was determined by comparison to cells transfected with the irrelevant control plasmid pBovine-shScrambled (Fig. 4B). Both vectors produced a very similar level of GAPDH knockdown, although not as effective as the GAPDH siRNAs. This is probably because the transfection efficiency for siRNAs is generally much greater than that of large plasmid vectors.

## Conclusion

We mined data from the bovine sequencing project and identified a bovine U6 snRNA promoter that is the putative bovine homologue of the human U6-8 snRNA promoter. This pol. III promoter sequence contains a number of functional sequence elements that are characteristic of this type of promoter and are essential for promoter function. The bovine U6 promoter sequence was used in the construction of plasmid based shRNA expression vectors pBovineU6-shEGFP and pBovineU6-shGapdh. These vectors efficiently induced RNAi in MDBK and Vero cells through production of shRNA molecules targeted at the exogenously expressed reporter gene EGFP, and the endogenous bovine gene GAPDH. The use of this promoter sequence and the shRNA vector cloning strategy described here will be advantageous in RNAi functional genomic experiments in bovine cells. The characterisation of this U6 promoter is an important step in the development of novel bovine species specific RNAi based therapeutics. For ethical reasons it is essential to minimise the introduction of non-bovine DNA sequences, consequently this research could be of significance in the transgenic delivery of shRNA molecules in bovine species.

## Methods

### Bovine U6 promoter isolation

Oligonucleotides synthesized in this study are indicated in Table 1. All the oligonucleotides were obtained from GeneWorks Pty Ltd, except for LL91 which was obtained from Proligo, and bovineGpdhPROBE from Applied Biosystems. The bovine BAC-end sequences deposited in the Genome Survey Sequence (GSS) section of GenBank, were compared with the U6 >95% identity sequence set for Rfam models RF00026  using BLAST with default parameters and a threshold e-value of 0.01 [33]. All hits detected were scored against the appropriate Rfam covariance model using the cmsearch function of INFERNAL [34]. Flanking repeat elements were identified using Repeatmasker with the minus cow option . Flanking sequences highly conserved between *Bos taurus *and the human genome sequence were identified using BLAT search (BLAST-like Alignment tool; UC-Santa Cruz genome server [35]) from the UC-Santa Cruz genome bioinformatics site .

A region of the bovine genome homologous to a bovine BAC-end sequence (GenBank Accession no. CC528275) predicted to contain a U6 snRNA promoter was amplified from *Bos taurus *genomic DNA isolated from whole blood using Wizard Genomic DNA Purification kit (Promega). First round PCR amplification used forward primer LL16 with a reverse primer TD66 designed from the highly conserved human U6 snRNA sequence. This PCR product was then used as template for semi-nested PCR with LL16 and antisense primer TD72 located 11 nt upstream of TD66 in the U6 snRNA sequence. The second round PCR produced a clean band of expected size (507 nt) that was gel purified using QIAquick gel extraction kit (Qiagen), ligated into pGEM-T Easy (Promega) as per the manufacturers instructions and sequenced.

### Expression vector construction and shRNA target sites

A bovine U6 EGFP shRNA construct (pBovineU6-shEGFP) was produced using a two-step PCR approach. The 1^st ^PCR paired LL16 with reverse primer LL23, comprising the last 20 nt of the promoter sequence, EGFP shRNA sense, loop, and 3 nt EGFP shRNA antisense sequence. This PCR product was used as template for semi-nested PCR to produce the full-length shRNA template, using LL16 and reverse primer LL13 with a short overlapping region, comprising the 4nt EGFP shRNA sense, loop, EGFP shRNA antisense, termination and *Xba*I.

A mouse U6 EGFP shRNA construct (pMouseU6-shEGFP) was produced using one-step PCR with p*Silencer *1.0-U6 siRNA Expression Vector (Ambion) as template. Universal primer M13 Forward was paired with reverse primer TD134, comprising the last 20 nt of the mouse promoter, and all other EGFP shRNA components.

The EGFP shRNA sequence used for both bovine and mouse vectors, had been shown previously to be effective in silencing gene expression [36]. A bovine U6 EGFP scrambled shRNA sequence (pBovineU6-shScrambled) was produced using a sequence that showed no significant homology to available monkey or bovine sequence data. Forward primer LL16 was paired with reverse primer LL31 comprising the last 20 nt of the bovine U6 promoter, and all other EGFP scrambled shRNA components.

Three siRNAs were designed based on the bovine GAPDH sequence obtained from GeneBank (Accession no. U85042) using the criteria described by Elbashir *et al*., 2002 (37). Target sites for bovine GAPDH were; GAPDH #1 (5'-AAGTTCAACGGCACAGTCAAG-3'); GAPDH #2 (5'-AACTTGACTGTGCCGTTGAAC-3'); GAPDH #3 (5'-AAGGTCATCCATGACCACTTT-3'). To generate siRNAs, oligonucleotides for sense and antisense-strands of each siRNA together with T7 promoter sites were synthesized and siRNAs were produced using the Ambion *Silencer *siRNA construction kit (Ambion). Oligonucleotides used for each were; GAPDH #1: LBG-as1 and LBG-as2; GAPDH #2: LBG-as3 and LBG-s4; GAPDH #3: LBG-as5 and LBG-s6 (Table 1).

A bovine U6 GAPDH shRNA expression construct (pBovineU6-shGapdh) was generated using a one-step PCR approach. LL16 was paired with LL05 which comprised the last 20 nt of the bovine U6 promoter and all other GAPDH shRNA sequence components including the GAPDH #1 siRNA sequence. Similarly, a mouse U6 GAPDH shRNA expression construct (pMouseU6-shGapdh) was also generated. M13 universal primer was paired with LL06, which also comprised the last 20 nt of the mouse U6 promoter and all other GAPDH shRNA components. All PCR products for shRNA expression constructs were ligated into pGEM-T Easy (Promega) and sequenced.

### Cell culture and transfection

MDBK (Madin Darby Bovine Kidney) and Vero (African Green monkey kidney) cell lines were cultured in Eagle's minimal essential medium (EMEM) medium containing 10% fetal calf serum (FCS), 2 mM glutamine, 10 mM HEPES, supplemented with penicillin (100 U/ml) and streptomycin (100 μg/ml). All cells were cultured in humidified atmosphere containing 5% CO_2 _at 37°C. Vero cells were grown to approximately 80% confluence on either 24-well plates (Nunc) for Flow Cytometry, on 8-well chamber slides (Lab-Tek) for fluorescence microscopy, or 6-well plates for shRNA detection. Cotransfection with 500 ng for 24-well plates and chamber slides, or 2.5 μg for 6-well plates of plasmid DNA (shRNA plasmids and/or pEGFP-N1 (Clonetech)) was carried out using Lipofectamine 2000 (Invitrogen) according to the manufacturer's instructions. MDBK cells were grown to 80–90% confluence in 75 cm^2 ^flasks (Corning), harvested and divided into aliquots of 1 × 10^6 ^cells per transfection. Approximately 2.5 μg of each plasmid was transfected per aliquot by electroporation using a Nucleofector (Amaxa) according to the manufacturer's instructions. Electroporated cells were dispensed into 8-well chamber slides for fluorescence microscopy, 6-well plates (Nunc) for flow cytometry or 24-well plates for Real-time PCR. Transfection of GAPDH siRNAs in MDBK cells grown to approximately 80% confluence on 24-well plates (Nunc) was achieved using Lipofectamine 2000 with approximately 10 nM of each siRNA.

### EGFP and GAPDH knockdown assays

EGFP expression was monitored at 72-hour post-transfection using fluorescence microscopy (Leica DMLB). Vero and MDBK cells cultured in 8-well chamber slides were washed with PBSA and mounting solution was applied (9:1 glycerol: PBSA). Cells to be analysed by flow cytometry were trypsinized and washed in PBSA. Cells were then resuspended in 200 μL 0.01% sodium azide and 2% FCS in PBSA and analysed using a FACScalibur (Becton Dickinson) flow cytometer. Data analysis was performed using CELLQuest software (Becton Dickinson).

Real-time RT-PCR reactions were carried out 48-hours post-transfection. MDBK cells transfected with GAPDH siRNAs and GAPDH shRNA vectors were harvest and cDNA was produced using the Cells-to-cDNA II kit (Ambion) using random hexamers. cDNA (2.5 μL) was added to a 22.5 μL Real-time RT -PCR master mix containing 12.5 μL 2X TaqMan Universal PCR Master Mix (Applied Biosystems), 2.5 μL (9 μM) each of sense primer BovineGpdhF and antisense primer bovineGpdhR, 2.5 μL (2.5 μM) of TaqMan MGB Probe (bovineGpdhPROBE) (Applied Biosystems) and 2.5 μL RNase-free water (Table 1). For RNA normalization, an 18s rRNA PCR was performed for each cDNA using the same PCR reagents except for the primers and probes which were the Eukaryotic 18srRNA Endogenous Control (Applied Biosystems). Real-time PCR was carried out in a 7700 Sequence Detection Instrument (Applied Biosystems) using the following thermal cycling profile: 95°C 1 min, followed by 35 cycles of amplification (95°C 15s, 61°C 30s, 68°C 30s).

### shRNA detection

Detection of EGFP shRNAs was carried by out using an RNAse protection assay on extracts enriched for small RNAs isolated from transfected Vero cells using a *mir*Vana miRNA Isolation Kit (Ambion). The RNA oligonucleotide LL91 was end labelled with [gamma-^32^P] ATP using a *mir*Vana Probe & Marker Kit (Ambion) and hybridised to the enriched RNAs using the *mir*Vana miRNA Detection Kit (Ambion). The RNA fragments were then separated by electrophoresis on a 15% denaturing polyacrylamide/ 8 M Urea gel and detected by autoradiography.

## Authors' contributions

LSL carried out all experiments and drafted the manuscript. TJD, RJM and MM participated in design and coordination of the research and revision of the manuscript. BPD and SM helped with bioinformatics and revision of the manuscript. All authors read and approved the final manuscript.

## Figures and Tables

**Table 1:** Synthesized oligonucleotides used in this study

Name	Sequence
LL16	5'-CCAGCAAAGCAACCGATTC-3'
TD66	5'-GGCCATGCTAATCTTCTCTG-3'
TD72	5'-TTTTAGTATATGTGCTGCCG-3'
LL23	5'-ATCTCTCTTGAAGATGAACTTCAGGGTCAGCGGTTTGCCTTTCGTCCACGG-3'
LL13	5'-TCTAGATTCCAAAAAAGCTGACCCTGAAGTTCATCTCTCTTGAAGATG-3'
TD134	5'-TCTAGATTCCAAAAAAGCTGACCCTGAAGTTCATCTCTCTTGAAGATGAACTTCAGGG TCAGCCAAACAAGGCTTTCTCCA-3'
LL31	5'-TCTAGATTCCAAAAAAAGCGCAGTGTTACTCCACTTCTCTTGAAAGTGGAGTAACACT GCGCTGGTTTGCCTTTCGTCCACGG-3'
LBG-as1	5'-AAGTTCAACGGCACAGTCAAGCCTGTCTC-3'
LBG-s2	5'-AACTTGACTGTGCCGTTGAACCCTGTCTC-3'
LBG-as3	5'-AAGGTCATCCATGACCACTTTCCTGTCTC-3'
LBG-s4	5'-AAAAAGTGGTCATGGATGACCCCTGTCTC-3'
LBG-as5	5'-AAGCTCATTTCCTGGTACGACCCTGTCTC-3'
LBG-s6	5'-AAGTCGTACCAGGAAATGAGCCCTGTCTC-3'
LL05	5'- TCTAGATTCCAAAAAAGTTCAACGGCACAGTCAAGTCTCTTGAACTTGACTGTGCCGT TGAACGGTTTGCCTTTCGTCCACGG-3'
LL06	5'-TCTAGATTCCAAAAAAGTTCAACGGCACAGTCAAGTCTCTTGAACTTGACTGTGCCGTT GAACCAAACAAGGCTTTCTCCA-3'
BovineGpdhF	5'-AACGACCACTTTGTCAAGCTCAT-3'
BovineGpdhR	5'-TCCACCACCCTGTTGCTGTA-3'
BovineGpdhPROBE	5'-TCCTGGTACGACAATGA-3'
LL91	5'-RGRAURGRARARCUURCRARGRGRGURCRARGRC-3'

'r' prefix to G, A and C represents ribonucleotide, and U indicates ribo-uridine.
